# Predictive value of the Ranson and BISAP scoring systems for the severity and prognosis of acute pancreatitis: A systematic review and meta-analysis

**DOI:** 10.1371/journal.pone.0302046

**Published:** 2024-04-30

**Authors:** Jianpeng Zhu, Linfei Wu, Yue Wang, Mengdie Fang, Qiang Liu, Xiaofeng Zhang

**Affiliations:** 1 The Fourth School of Clinical Medicine, Zhejiang Chinese Medical University, Hangzhou, Zhejiang, China; 2 Zhejiang University of Medicine, Hangzhou, Zhejiang, China; 3 Department of Gastroenterology, Affiliated Hangzhou First People’s Hospital, Zhejiang University School of Medicine, Hangzhou, Zhejiang, China; Sapporo Medical University, JAPAN

## Abstract

**Background:**

To systematically assess and compare the predictive value of the Ranson and Bedside Index of Severity in Acute Pancreatitis (BISAP) scoring systems for the severity and prognosis of acute pancreatitis (AP).

**Methods:**

PubMed, Embase, Cochrane Library, and Web of Science were systematically searched until February 15, 2023. Outcomes in this analysis included severity and prognosis [mortality, organ failure, pancreatic necrosis, and intensive care unit (ICU) admission]. The revised Quality Assessment of Diagnostic Accuracy Studies (QUADAS-2) tool was used to evaluate the quality of diagnostic accuracy studies. The threshold effect was evaluated for each outcome. The sensitivity, specificity, positive likelihood ratio (PLR), negative likelihood ratio (NLR), diagnostic odds ratio (DOR), and the area under the summary receiver operating characteristic (SROC) curve (AUC) as well as 95% confidence intervals (CI) were calculated. The DeLong test was used for AUC comparisons. For the outcome evaluated by over 9 studies, publication bias was assessed using the Deeks’ funnel plot asymmetry test.

**Results:**

Totally 17 studies of 5476 AP patients were included. For severity, the pooled sensitivity of the Ranson and BISAP was 0.95 (95%CI: 0.87, 0.98) and 0.67 (95%CI: 0.27, 0.92); the pooled specificity of the Ranson and BISAP was 0.74 (0.52, 0.88) and 0.95 (95%CI: 0.85, 0.98); the pooled AUC of the Ranson and BISAP was 0.95 (95%CI: 0.93, 0.97) and 0.94 (95%CI: 0.92, 0.96) (*P* = 0.480). For mortality, the pooled sensitivity of the Ranson and BISAP was 0.89 (95%CI: 0.73, 0.96) and 0.77 (95%CI: 0.58, 0.89); the pooled specificity of the Ranson and BISAP was 0.79 (95%CI: 0.68, 0.87) and 0.90 (95%CI: 0.86, 0.93); the pooled AUC of the Ranson and BISAP was 0.91 (95%CI: 0.88, 0.93) and 0.92 (95%CI: 0.90, 0.94) (*P* = 0.480). For organ failure, the pooled sensitivity of the Ranson and BISAP was 0.84 (95%CI: 0.76, 0.90) and 0.78 (95%CI: 0.60, 0.90); the pooled specificity of the Ranson and BISAP was 0.84 (95%CI: 0.63, 0.94) and 0.90 (95%CI: 0.72, 0.97); the pooled AUC of the Ranson and BISAP was 0.86 (95%CI: 0.82, 0.88) and 0.90 (95%CI: 0.87, 0.93) (*P* = 0.110). For pancreatic necrosis, the pooled sensitivity of the Ranson and BISAP was 0.63 (95%CI: 0.35, 0.84) and 0.63 (95%CI: 0.23, 0.90); the pooled specificity of the Ranson and BISAP was 0.90 (95%CI: 0.77, 0.96) and 0.93 (95%CI: 0.89, 0.96); the pooled AUC of the Ranson and BISAP was 0.87 (95%CI: 0.84, 0.90) and 0.93 (95%CI: 0.91, 0.95) (*P* = 0.001). For ICU admission, the pooled sensitivity of the Ranson and BISAP was 0.86 (95%CI: 0.77, 0.92) and 0.63 (95%CI: 0.52, 0.73); the pooled specificity of the Ranson and BISAP was 0.58 (95%CI: 0.55, 0.61) and 0.84 (95%CI: 0.81, 0.86); the pooled AUC of the Ranson and BISAP was 0.92 (95%CI: 0.81, 1.00) and 0.86 (95%CI: 0.67, 1.00) (*P* = 0.592).

**Conclusion:**

The Ranson score was an applicable tool for predicting severity and prognosis of AP patients with reliable diagnostic accuracy in resource and time-limited settings. Future large-scale studies are needed to verify the findings.

## Introduction

Acute pancreatitis (AP), an inflammatory disease of the pancreas, is one of the most common gastrointestinal diseases which requires acute hospital admission, with a mounting incidence, significant morbidity and succeeding mortality [1–3]. This disorder exhibits varying severity [4]. The revised Atlanta Classification and Definitions in 2012 classifies AP as mild, moderately severe and severe acute [5]. The prognosis of AP patients primarily depends on the progress of organ failure and secondary infection of pancreatic or peripancreatic necrosis [6]. Besides, patients with AP usually need intensive care unit (ICU) admission, particularly as signs of multi-organ failure appear [7]. Early severity stratification and prognostic prediction are essential for lowering the mortality rate of AP patients [8].

There are various scoring systems to evaluate the severity and outcomes of AP. The Ranson score, developed in 1974, is the first scoring system to predict AP, which has been criticized for its low predictive ability and delayed management despite continuous wide application [9, 10]. The Bedside Index of Severity in Acute Pancreatitis (BISAP) is a commonly used scoring system at present, and compared with the Ranson, the BISAP can be adopted at admission and has fewer parameters [11]. A review by Ong and Shelat shows that the Ranson score consistently exhibits similar predictive accuracy to the BISAP scoring system, advocating for the sustained clinical practicability of the Ranson score in modern times [9]. The Acute Physiology, and Chronic Health Examination II (APACHE II) has been reported to be the most accurate scoring system for predicting mortality [12]. It is the most widely utilized mortality prediction score among critically ill patients, but it had 12 items with many clinical parameters, so its application may be cumbersome which limits its widespread use. Besides, the APACHE II is devised for patients admitted to the ICU and is therefore not suitable for early prediction of the severity of AP. The BISAP and Ranson were shown to have overlapped AUCs with the APACHE II [13]. The BISAP and Ranson are determined upon admission or within 48 hours of admission, but in the computed tomography severity index (CTSI) prediction, the recommended timing for CT examination is 72 to 96 hours after symptom onset [14, 15]. This may limit the early predictive ability of CTSI, as the BISAP and Ranson can predict severity or mortality early with similar performances [12]. Additionally, the presence of inter-observer variability can affect the accuracy of CTSI score calculation [9]. For these reasons, this study paid attention to the early prediction of AP severity and outcomes with the Ranson and BISAP. Several studies have comprehensively evaluated the predictive performance of the BISAP for severity and prognosis in AP [16–18], whereas no specific meta-analysis is conducted for direct comparison between the Ranson and BISAP. Given that the Ranson does not consider imaging data, uses multiple parameters, and misses possibly valuable window for early treatment, whether it is still applicable requires comprehensive quantitative evaluation.

This systematic review and meta-analysis aimed to systematically assess and compare the predictive value of the Ranson and BISAP scoring systems for the severity and prognosis of AP based on the revised Atlanta Classification and Definitions in 2012.

## Methods

### Search strategy

PubMed, Embase, Cochrane Library, and Web of Science were systematically searched by two independent authors (Y Wang, MD Fang). The last search was performed on February 15, 2023. English search terms included “Pancreatitis” OR “Pancreatitides” OR “Acute Pancreatitis” OR “Acute Pancreatitides” OR “Pancreatic Parenchymal Edema” OR “Pancreatic Parenchyma with Edema” OR “Peripancreatic Fat Necros*” AND “Ranson” OR “BISAP” OR “Bedside Index for Severity In Acute Pancreatitis”. Endnote X9 (Clarivate, Philadelphia, PA, USA) was applied for primary screening based on titles and abstracts of the retrieved studies, followed by screening according to full texts. Disagreements were settled by another author (JP Zhu) to reach a consensus. This systematic review and meta-analysis was conducted following the reporting guidelines of Meta-analysis of Observational Studies in Epidemiology (MOOSE).

### Eligibility criteria

#### Inclusion criteria

(a) studies on patients with AP of varying severity [5]; (b) studies reporting the BISAP versus Ranson with a cutoff point at 3 [17, 19]; (c) studies on at least one of the following outcomes: severity, mortality, organ failure, pancreatic necrosis, and ICU admission; (d) observational studies; (e) studies providing relevant data to calculate indicators such as sensitivity and specificity or area under the curve (AUC) values; (f) English literature; (g) studies published since 2012.

#### Exclusion criteria

(a) studies on patients with chronic or recurrent pancreatitis, pregnant or lactating patients, or patients who had been hospitalized for less than 48 hours; (b) studies reporting the BISAP versus Ranson with an unclear cutoff point or a cutoff point that was not 3; (c) meta-analyses, reviews, meeting abstracts, animal experiments, case reports, letters, or comments.

### Outcome measures

Outcomes in this analysis included severity and prognosis (mortality, organ failure, pancreatic necrosis, and ICU admission). Severity was defined as persistent single or multiple organ failure for more than 48 h. Organ failure was defined as two or more points in one of the cardiovascular, renal and respiratory systems in the modified Marshall scoring system. Pancreatic necrosis was defined as the absence of enhanced pancreatic parenchyma on contrast-enhanced computed tomography (CECT).

### Data extraction and quality assessment

Two authors (Y Wang, MD Fang) independently collected data from eligible studies, including the first author, year of publication, author’s country, study period, study design, sample size (N), sex (male/female), age (years), etiology, and endpoints. Disagreements were resolved by discussion with a third author (LF Wu). The revised Quality Assessment of Diagnostic Accuracy Studies (QUADAS-2) tool was used to evaluate the quality of diagnostic accuracy studies, based on the risk of bias and clinical applicability [20]. The risk of bias includes patient selection, index test, reference standard, and flow and timing. Clinical applicability includes patient selection, index test, and reference standard. Each item was classified as high (risk), low (risk), or unclear (risk).

### Statistical analysis

Statistical analysis was carried out by Meta-disc 1.4 (Clinical Biostatistics, Ramony Cajal Hospital, Madrid, Spain), Stata 15.1 (Stata Corporation, College Station, Texas, USA), and Revman 5.4 (The Nordic Cochrane Centre, The Cochrane Collaboration, Copenhagen, Denmark). Results were obtained through direct extraction or indirect calculation. Meta-disc 1.4 was adopted to determine whether there was a threshold effect. When the Spearman correlation coefficient between the logarithm of sensitivity and the logarithm of 1-specificity showed a strong positive correlation, it indicated the existence of a threshold effect. To measure the predictive performance of the Ranson and BISAP, Stata 15.1 was employed to assess the sensitivity, specificity, positive likelihood ratio (PLR), negative likelihood ratio (NLR), and diagnostic odds ratio (DOR) as well as 95% confidence intervals (CI) for clinical outcomes using a bivariate model. Summary receiver operating characteristic (SROC) curves were generated, and the AUC was calculated with 95%CI. The DeLong test was used for AUC comparisons. For the outcome evaluated by over 9 studies, publication bias was assessed using the Deeks’ funnel plot asymmetry test via Stata 15.1. Publication bias was neglected when a funnel plot was symmetric. Revman 5.4 was applied to draw a quality assessment chart for the included studies. *P*<0.05 was deemed as statistically significant differences.

## Results

### Study selection

**Fig 1:**
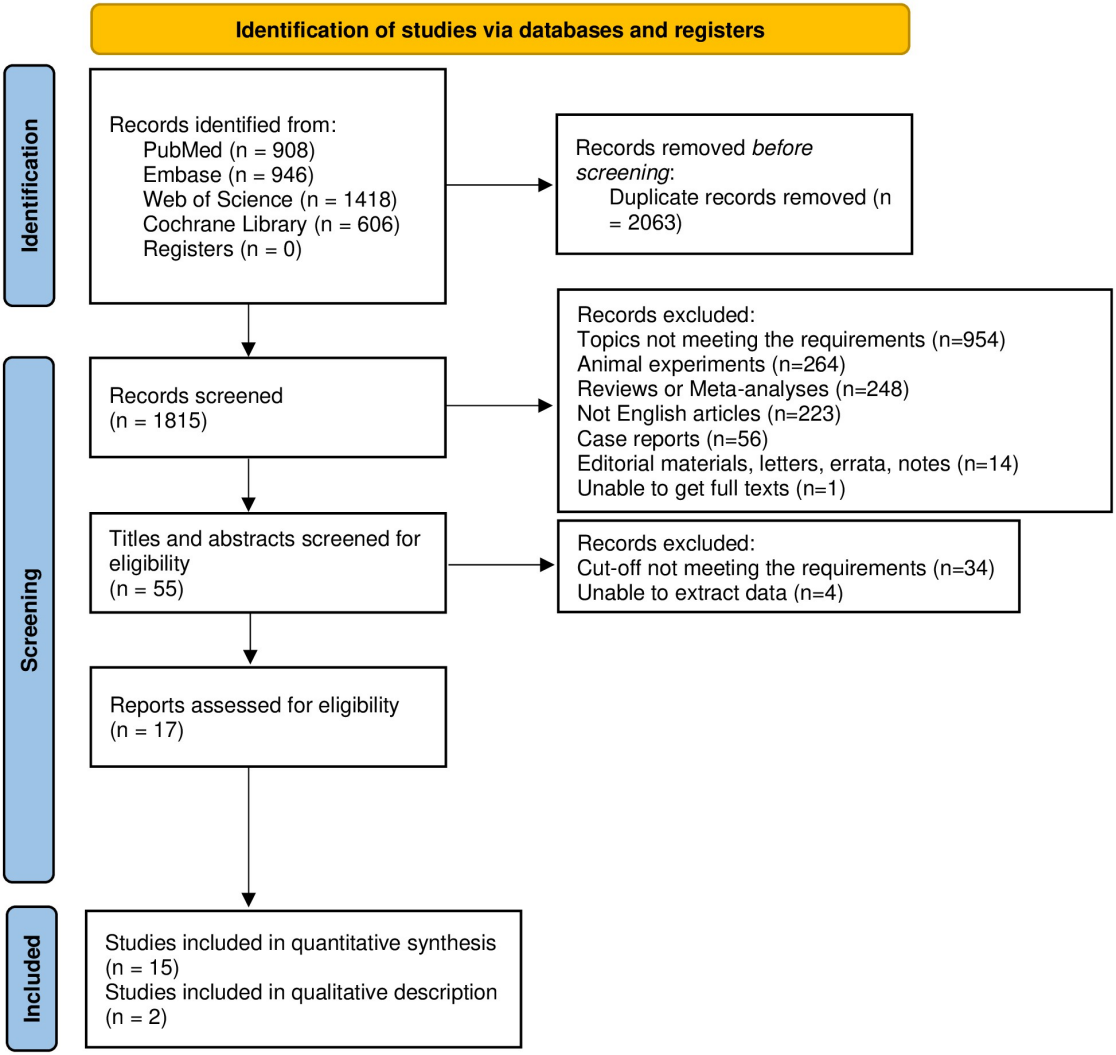
Selection process of qualified studies.

A total of 3878 studies were identified, with 908 from PubMed, 946 from Embase, 606 Cochrane Library, and 1418 from Web of Science. After removing duplicates, 1815 studies were included for screening based on titles and abstracts. Then remaining 55 studies were subject to full-text screening. Ultimately, 17 [21–37] studies of 5476 AP patients were eligible for this systematic review and meta-analysis, with 15 studies included for quantitative analysis. Fig 1 presents the selection process of qualified studies.

### Characteristics of the included studies

Among the included studies, 4 studies came from China, 4 from India, 4 from Korea, 3 from Pakistan, 1 from Serbia, and 1 from Singapore. The year of publication ranged from 2013 to 2022. Eight articles were designed as prospective studies, and 9 as retrospective studies. The characteristics of the included studies are shown in Table 1.

**Table 1:** Characteristics of the included studies.

Author	Year	Country	Study period	Study design	N	Sex (M/F)	Age (years)	Etiology	Endpoints
Athavale [37]	2022	India	2020–2022	Prospective	100	81/19	18–65	Alcoholic 75, biliary duct obstruction 20, other 5	Mortality, pancreatic necrosis
Zhao [36]	2022	China	2018–2020	Retrospective	284	161/123	57.26±18.12	Gallstone 154, hyperlipidemia 69, alcoholic 13, other 61	Mortality
Kapadia [26]	2021	Pakistan	2017–2018	Retrospective	136	88/48	42.04±16.42	-	Mortality
Teng [31]	2021	Singapore	2009–2016	Retrospective	653	383/270	58.7 ± 17.5	Gallstone 404, alcoholic 38, idiopathic 61, hypertriglyceridemia 19, autoimmune 4, hypercalcemia 3, drug induced 6, other 47	Severity, mortality, ICU admission
Wu [32]	2021	China	2003–2020	Retrospective	1848	1260/588	48.22±16.21	Gallstone 711, alcoholic 199, hypertriglyceridaemia 309	Mortality
Yan [34]	2021	China	2018–2020	Retrospective	465	253/212	54.6 (22–85)	Gallstone 171, hypertriglyceridaemia 122, alcoholic 57, post-ERCP 12, other 16, unknown 87	Organ failure, ICU admission
Arif [21]	2019	Pakistan	2015	Prospective	206	81/125	35.25±8.29	-	Severity
Cho [23]	2019	Korea	2015–2018	Prospective	269	179/90	57.6±18.6	Gallstone 128, alcoholic 93, hypertriglyceridemia 16, idiopathic 32	Severity
Hagjer [24]	2018	India	2015–2016	Prospective	60	41/19	37.17 ± 11.77	Alcoholic 27, gallstone 24, idiopathic 9	Mortality, organ failure, pancreatic necrosis
Harshit Kumar [25]	2018	India	2015–2016	Prospective	50	17/33	48.4 (19–80)	Gallstone 37, alcoholic 9, idiopathic 3, traumatic 1	Organ failure, pancreatic necrosis, ICU admission
Spasić [30]	2017	Serbia	2011–2014	Prospective	132	84/48	23–86	Gallstone 68, alcoholic 33, other 31	Mortality
Lee [27]	2016	Korea	2010–2013	Prospective	146	92/54	50.6±18.3	Biliary 72, alcoholic 52, hypertriglyceridemia 7, idiopathic 15	Severity
Yadav [33]	2016	India	2012–2014	Prospective	119	84/35	38.94±14.59	Alcoholic 48, gallstone 37, other 34	Severity, mortality, pancreatic necrosis
Yang [35]	2016	China	2007–2015	Retrospective	326	184/142	44 (14–85)	Hyperlipidemic	Mortality
Shabbir [29]	2015	Pakistan	2010	Retrospective	80	35/45	46.86 ± 15.75	-	Mortality
Cho [22]	2013	Korea	2008–2010	Retrospective	299	208/91	52.1±16.4	Alcoholic 128, gallstone 76, idiopathic 69, post-ERCP 5, drug 5, other 16	Severity, mortality
Park [28]	2013	Korea	2007–2010	Retrospective	303	216/87	52±17	Alcoholic 151, biliary 88, idiopathic 49, cancer 11, hypertriglyceridemia 4	Mortality, organ failure, pancreatic necrosis

N, sample size; M/F, male/female; ICU, intensive care unit.

### Quality assessment

For the risk of bias, 16 studies [22–37] had low risks, and 1 study [21] exhibited a high risk in the patient selection; most studies had unclear risks in the index test; 6 studies [24–26, 28, 32, 37] showed high risks, and 11 [21–23, 27, 29–31, 33–36] had low risks in the reference standard; all the included studies had low risks in the flow and timing. For clinical applicability, all studies except 1 study [35] had low risks in the patient selection; 6 studies [23, 26, 29, 30, 34, 36] had high risks, and 11 studies [21, 22, 24, 25, 27, 28, 31–33, 35, 37] had low risks in the index test; 8 studies [24–26, 28, 30, 32, 35, 37] had high risks, and 9 [21–23, 27, 29, 31, 33, 34, 36] showed low risks in the reference standard (Table 2).

**Table 2:** Methodological quality assessment of the included studies.

Study	Risk of bias				Applicability		
	Patient Selection	Index Test	Reference Standard	Flow and Timing	Patient Selection	Index Test	Reference Standard
Athavale.2022 [37]	L	U	H	L	L	L	H
Zhao.2022 [36]	L	H	L	L	L	H	L
Kapadia.2021 [26]	L	L	H	L	L	H	H
Teng.2021 [31]	L	U	L	L	L	L	L
Wu.2021[32]	L	H	H	L	L	L	H
Yan.2021 [34]	L	U	L	L	L	H	L
Arif.2019 [21]	H	L	L	L	L	L	L
Cho.2019 [23]	L	U	L	L	L	H	L
Hagjer.2018 [24]	L	U	H	L	L	L	H
Harshit Kumar.2018 [25]	L	U	H	L	L	L	H
Spasić.2017 [30]	L	U	L	L	L	H	H
Lee.2016 [27]	L	U	L	L	L	L	L
Yadav.2016 [33]	L	U	L	L	L	L	L
Yang.2016 [35]	L	U	L	L	H	L	H
Shabbir.2015 [29]	L	L	L	L	L	H	L
Cho.2013 [22]	L	U	L	L	L	L	L
Park.2013 [28]	L	U	H	L	L	L	H

L, low risk; H, high risk; U, unclear risk.

### Predictive performance of the Ranson and BISAP for severity

**Fig 2:**
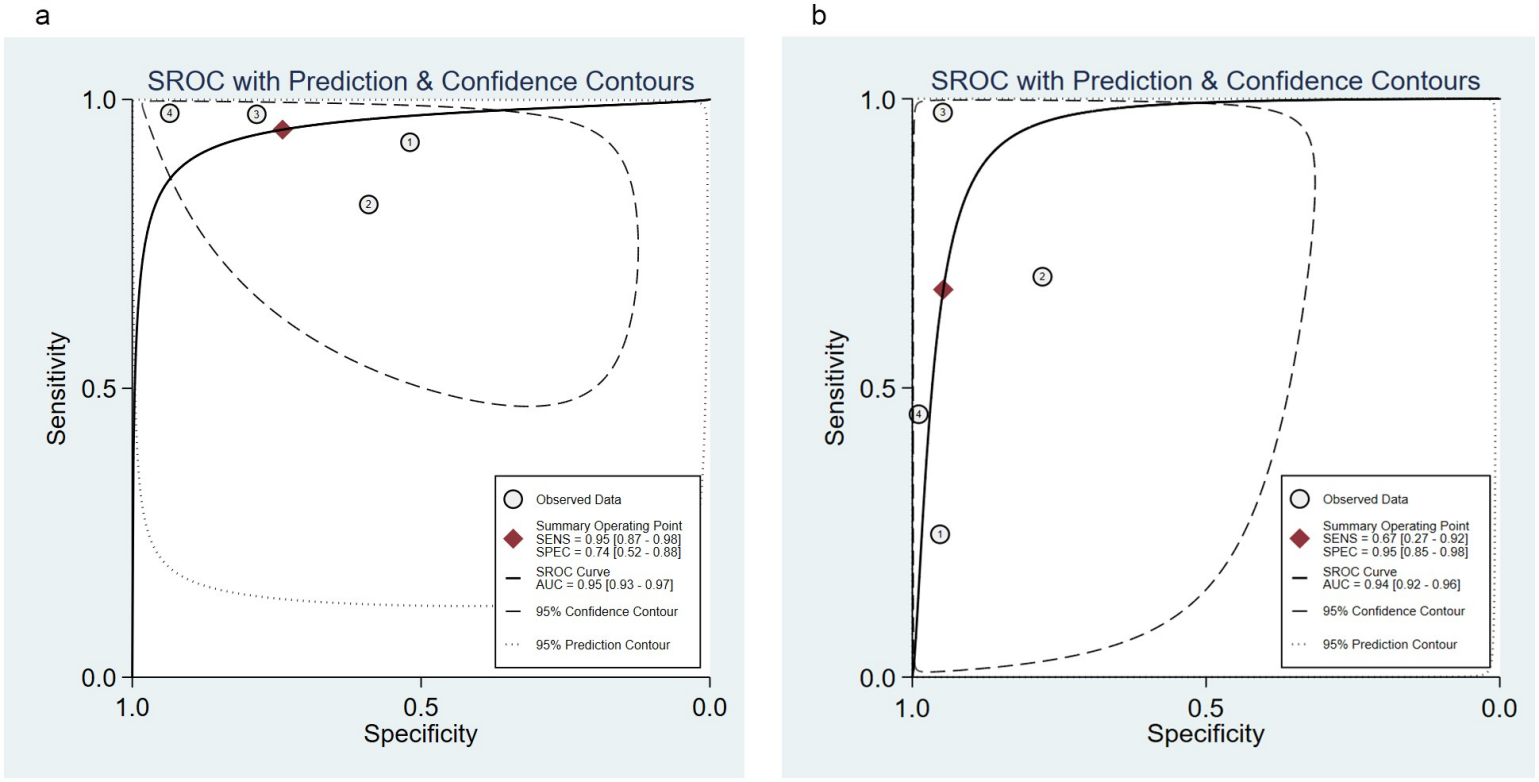
SROC curve of the (a) Ranson and (b) BISAP for predicting severity in AP. SROC, Summary receiver operating characteristic; BISAP, Bedside Index of Severity in Acute Pancreatitis; AP, acute pancreatitis.

Four studies [21, 22, 31, 33] provided data on the Ranson and BISAP for severity. The SROC curves of the Ranson and BISAP did not present “shoulder-arm” distributions, indicating no threshold effects (Fig 2). Further, the Spearman correlation coefficient for the Ranson and BISAP was -0.8 (*P* = 0.2) and 0.6 (*P* = 0.4), respectively, which confirmed the absence of threshold effects.

For the Ranson, the pooled sensitivity was 0.95 (95%CI: 0.87, 0.98); the pooled specificity was 0.74 (0.52, 0.88); the pooled PLR was 3.64 (95%CI: 1.74, 7.62); the pooled NLR was 0.07 (95%CI: 0.02, 0.21); the pooled DOR was 51.66 (95%CI: 9.28, 287.66), and the pooled AUC was 0.95 (95%CI: 0.93, 0.97). For the BISAP, the pooled sensitivity was 0.67 (95%CI: 0.27, 0.92); the pooled specificity was 0.95 (95%CI: 0.85, 0.98); the pooled PLR was 12.71 (95%CI: 4.07, 39.69); the pooled NLR was 0.35 (95%CI: 0.11, 1.09); the pooled DOR was 36.50 (95%CI: 5.57, 239.39), and the pooled AUC was 0.94 (95%CI: 0.92, 0.96). No significant difference was found in the pooled AUC between the Ranson and BISAP (*P* = 0.480) (Table 3, Fig 2, S1 Fig).

**Table 3:** Pooled results of the Ranson and BISAP for predicting the severity and prognosis of AP.

Score	SEN	SPE	PLR	NLR	DOR	AUC	*P* (AUC)	Threshold effect
Severity								
Ranson	0.95 (0.87, 0.98)	0.74 (0.52, 0.88)	3.64 (1.74, 7.62)	0.07 (0.02, 0.21)	51.66 (9.28, 287.66)	0.95 (0.93, 0.97)	0.480	r = -0.8, *P* = 0.2
BISAP	0.67 (0.27, 0.92)	0.95 (0.85, 0.98)	12.71 (4.07, 39.69)	0.35 (0.11, 1.09)	36.50 (5.57, 239.39)	0.94 (0.92, 0.96)		r = 0.6, *P* = 0.4
Mortality								
Ranson	0.89 (0.73, 0.96)	0.79 (0.68, 0.87)	4.22 (2.74, 6.50)	0.14 (0.05, 0.36)	30.37 (10.69, 86.29)	0.91 (0.88, 0.93)	0.480	r = 0.516, *P* = 0.086
BISAP	0.77 (0.58, 0.89)	0.90 (0.86, 0.93)	8.00 (5.56, 11.50)	0.25 (0.13, 0.50)	31.59 (13.56, 73.58)	0.92 (0.90, 0.94)		r = 0.427, *P* = 0.167
Organ failure								
Ranson	0.84 (0.76, 0.90)	0.84 (0.63, 0.94)	5.18 (1.99, 13.53)	0.19 (0.11, 0.31)	27.40 (7.41, 101.33)	0.86 (0.82, 0.88)	0.110	r = 0.000, *P* = 1.000
BISAP	0.78 (0.60, 0.90)	0.90 (0.72, 0.97)	7.64 (3.01, 19.41)	0.24 (0.13, 0.44)	31.64 (15.69, 63.83)	0.90 (0.87, 0.93)		r = 0.949, *P* = 0.051
Pancreatic necrosis								
Ranson	0.63 (0.35, 0.84)	0.90 (0.77, 0.96)	6.04 (1.78, 20.54)	0.42 (0.19, 0.92)	14.48 (2.02, 103.94)	0.87 (0.84, 0.90)	0.001	r = -0.8, *P* = 0.104
BISAP	0.63 (0.23, 0.90)	0.93 (0.89, 0.96)	8.97 (3.98, 20.19)	0.40 (0.13, 1.19)	22.39 (3.64, 137.79)	0.93 (0.91, 0.95)		r = -0.6, *P* = 0.285
ICU admission								
Ranson	0.86 (0.77, 0.92)	0.58 (0.55, 0.61)	2.93 (1.43, 6.00)	0.23 (0.14, 0.38)	26.80 (5.31, 135.23)	0.92 (0.81, 1.00)	0.592	r = 1.000, *P* = 0.000
BISAP	0.63 (0.52, 0.73)	0.84 (0.81, 0.86)	3.50 (1.70, 7.24)	0.47 (0.21, 1.07)	7.68 (2.49, 23.74)	0.86 (0.67, 1.00)		r = 0.5, *P* = 0.667

*P* (AUC): *P* value for AUC.

AP, acute pancreatitis; AUC, area under curve; DOR, diagnostic odds ratio; NLR, negative likelihood ratio; PLR, positive likelihood ratio; SEN, sensitivity; SPE, specificity; BISAP, Bedside Index of Severity in Acute Pancreatitis; ICU, intensive care unit.

Cho et al. [27] showed that the AUC of the Ranson and BISAP was 0.848 (95%CI: 0.77–0.92) and 0.826 (95%CI: 0.74–0.92), respectively. In the study of Lee et al. [27], the AUC of the Ranson and BISAP was 0.76 (95%CI: 0.64–0.87) and 0.66 (95%CI: 0.50–0.82), respectively.

### Predictive performance of the Ranson and BISAP for mortality

**Fig 3:**
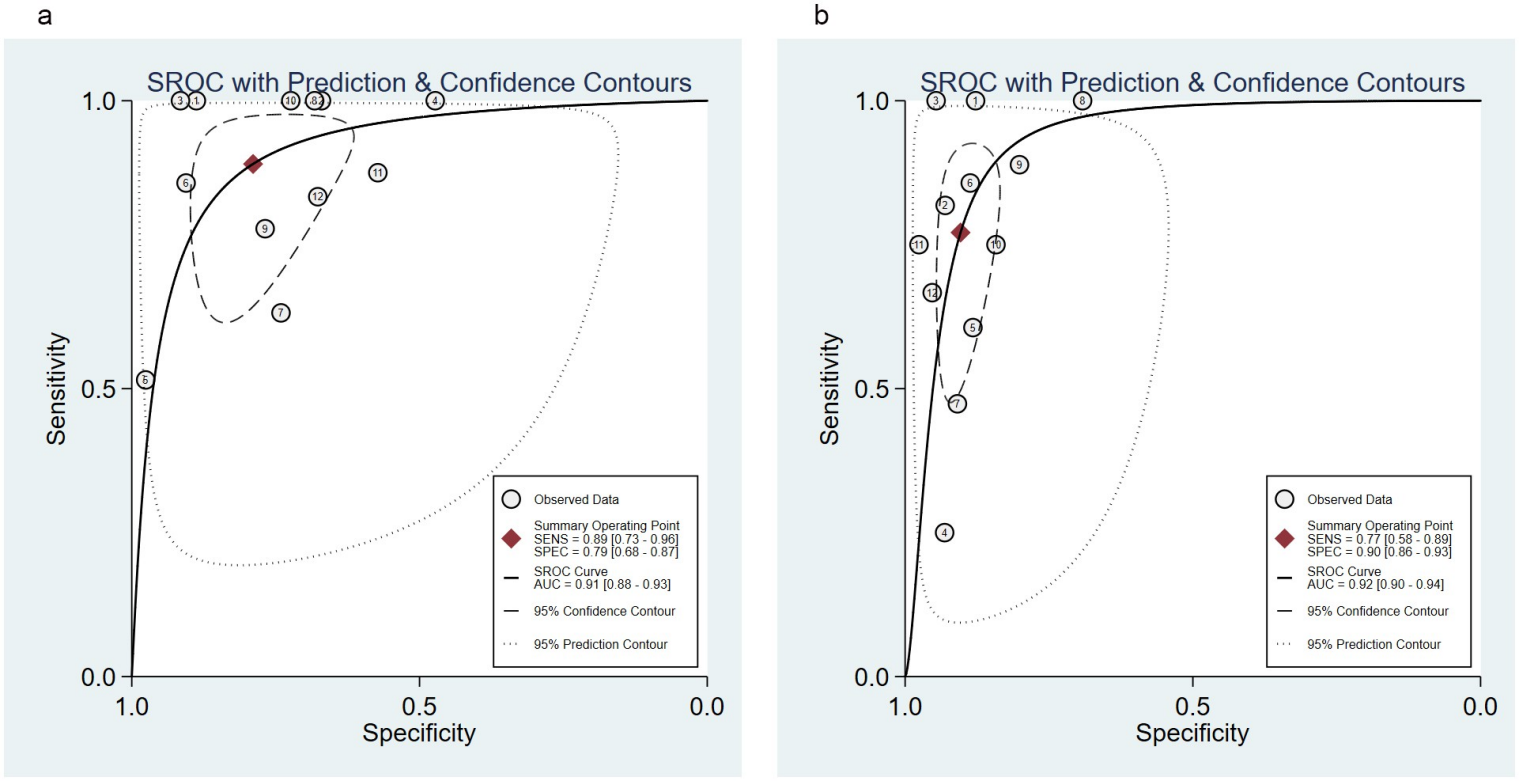
SROC curve of the (a) Ranson and (b) BISAP for predicting mortality in AP. SROC, Summary receiver operating characteristic; BISAP, Bedside Index of Severity in Acute Pancreatitis; AP, acute pancreatitis.

Comparison of the Ranson and BISAP for mortality was assessed by 12 studies [22, 23, 26, 28–33, 35–37]. No threshold effects were shown according to no “shoulder-arm” distributions in the SROC curves (Fig 3). The Spearman correlation coefficient for the Ranson and BISAP was 0.516 (*P* = 0.086) and 0.427 (*P* = 0.167), respectively, further suggesting the absence of threshold effects.

For the Ranson, the pooled sensitivity was 0.89 (95%CI: 0.73, 0.96); the pooled specificity was 0.79 (95%CI: 0.68, 0.87); the pooled PLR was 4.22 (95%CI: 2.74, 6.50); the pooled NLR was 0.14 (95%CI: 0.05, 0.36); the pooled DOR was 30.37 (95%CI: 10.69, 86.29), and the pooled AUC was 0.91 (95%CI: 0.88, 0.93). For the BISAP, the pooled sensitivity was 0.77 (95%CI: 0.58, 0.89); the pooled specificity was 0.90 (95%CI: 0.86, 0.93); the pooled PLR was 8.00 (95%CI: 5.56, 11.50); the pooled NLR was 0.25 (95%CI: 0.13, 0.50); the pooled DOR was 31.59 (95%CI: 13.56, 73.58), and the pooled AUC was 0.92 (95%CI: 0.90, 0.94). No significant difference was found in the pooled AUC between the Ranson and BISAP (*P* = 0.480) (Table 3, Fig 3, S2 Fig).

### Predictive performance of the Ranson and BISAP for organ failure

**Fig 4:**
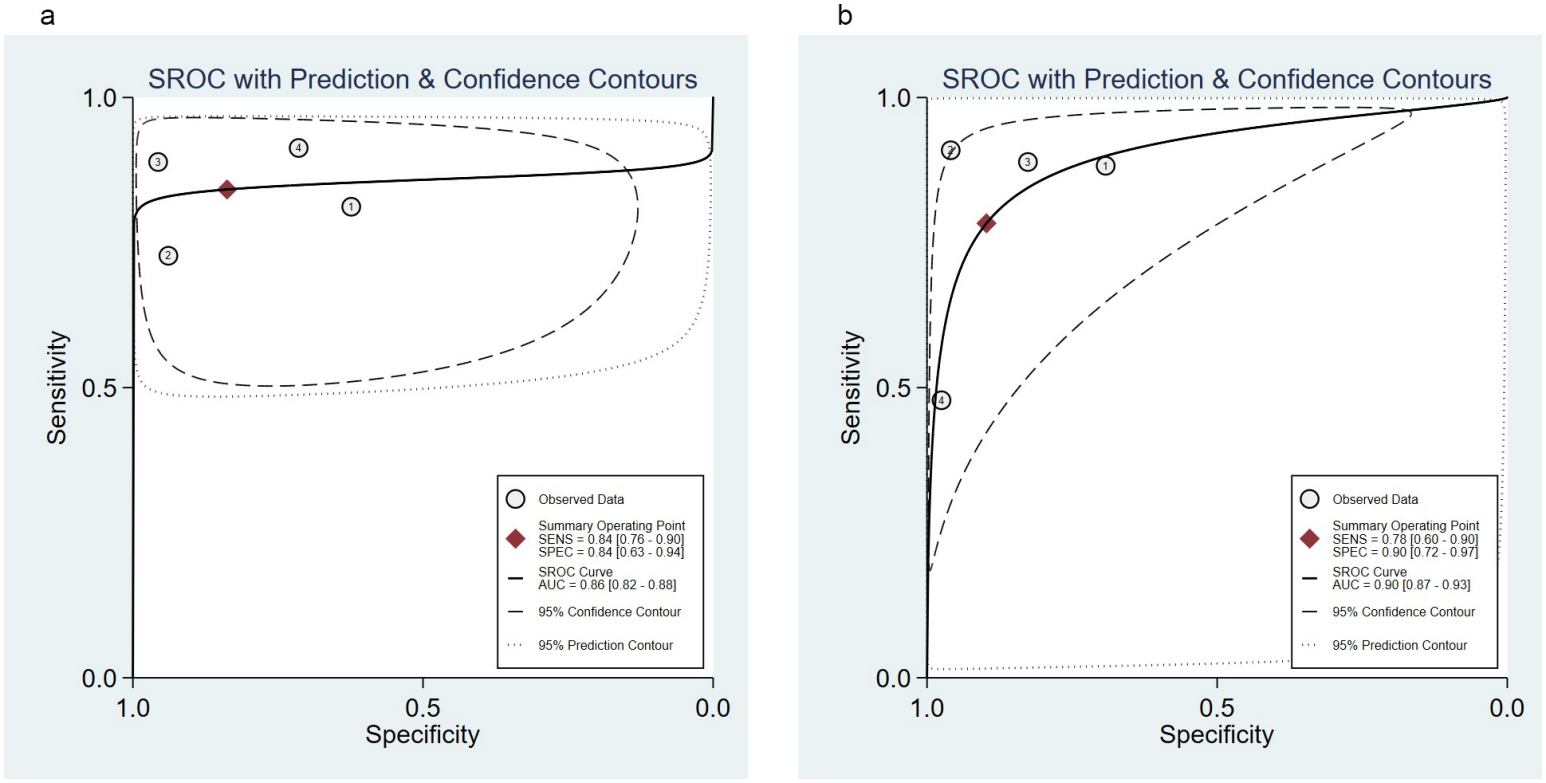
SROC curve of the (a) Ranson and (b) BISAP for predicting organ failure in AP. SROC, Summary receiver operating characteristic; BISAP, Bedside Index of Severity in Acute Pancreatitis; AP, acute pancreatitis.

Information about organ failure was shown in 4 studies [24, 25, 28, 34]. No “shoulder-arm” distributions in the SROC curve illustrated that there were no threshold effects (Fig 4). The Spearman correlation coefficient for the Ranson and BISAP was 0.000 (*P* = 1.000) and 0.949 (*P* = 0.051), respectively, confirming no threshold effects.

For the Ranson, the pooled sensitivity was 0.84 (95%CI: 0.76, 0.90); the pooled specificity was 0.84 (95%CI: 0.63, 0.94); the pooled PLR was 5.18 (95%CI: 1.99, 13.53); the pooled NLR was 0.19 (95%CI: 0.11, 0.31); the pooled DOR was 27.40 (95%CI: 7.41, 101.33), and the pooled AUC was 0.86 (95%CI: 0.82, 0.88). For the BISAP, the pooled sensitivity was 0.78 (95%CI: 0.60, 0.90); the pooled specificity was 0.90 (95%CI: 0.72, 0.97); the pooled PLR was 7.64 (95%CI: 3.01, 19.41); the pooled NLR was 0.24 (95%CI: 0.13, 0.44); the pooled DOR was 31.64 (95%CI: 15.69, 63.83), and the pooled AUC was 0.90 (95%CI: 0.87, 0.93). No significant difference was found in the pooled AUC between the Ranson and BISAP (*P* = 0.110) (Table 3, Fig 4, S3 Fig).

### Predictive performance of the Ranson and BISAP for pancreatic necrosis

**Fig 5:**
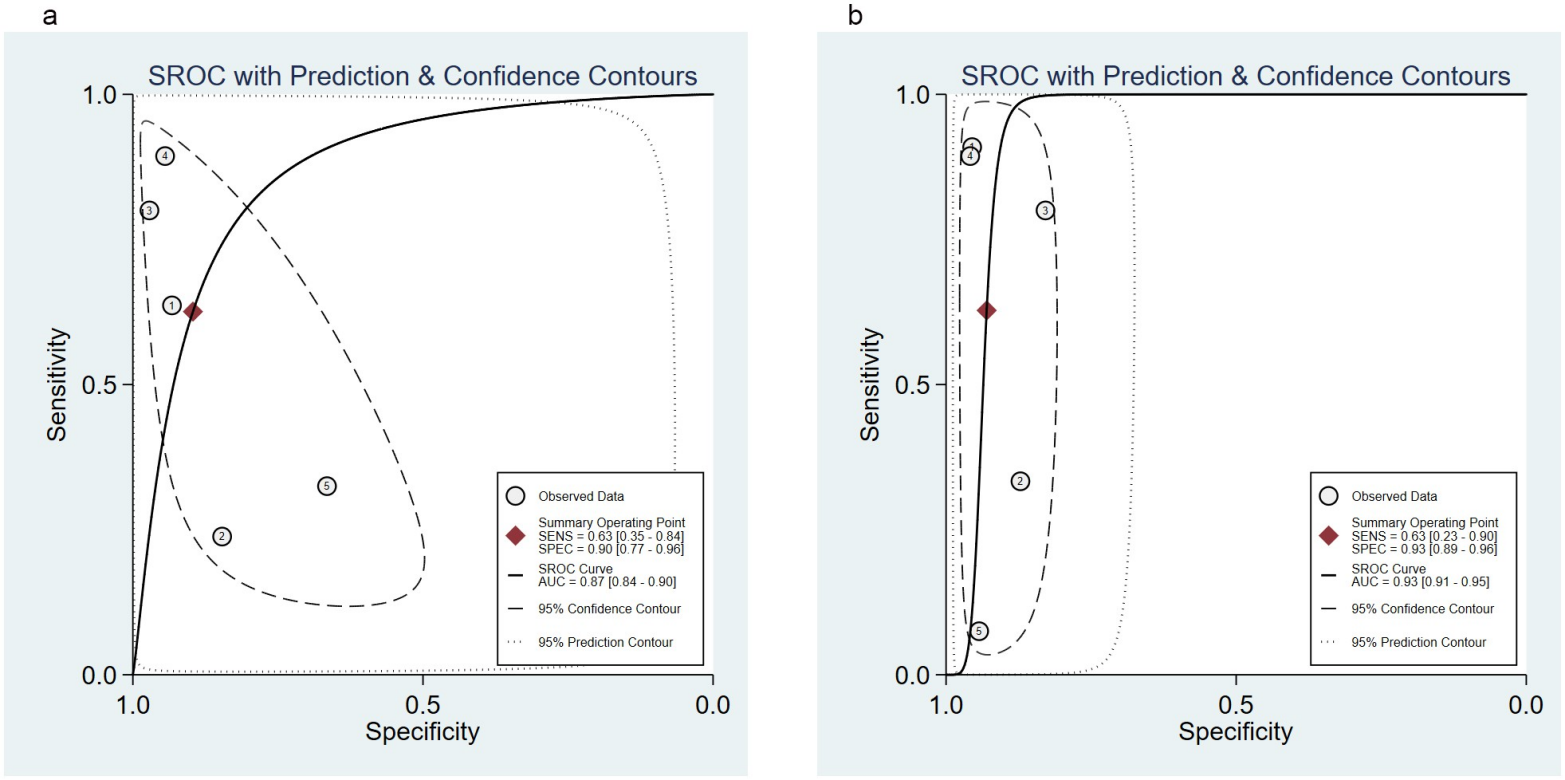
SROC curve of the (a) Ranson and (b) BISAP for predicting pancreatic necrosis in AP. SROC, Summary receiver operating characteristic; BISAP, Bedside Index of Severity in Acute Pancreatitis; AP, acute pancreatitis.

Five studies [24, 25, 28, 33, 37] evaluated the Ranson and BISAP for pancreatic necrosis. The SROC curves did not show “shoulder-arm” distributions, suggesting no threshold effects (Fig 5). The Spearman correlation coefficient for the Ranson and BISAP was -0.8 (*P* = 0.104) and -0.6 (*P* = 0.285), respectively, further indicating the absence of threshold effects.

For the Ranson, the pooled sensitivity was 0.63 (95%CI: 0.35, 0.84); the pooled specificity was 0.90 (95%CI: 0.77, 0.96); the pooled PLR was 6.04 (95%CI: 1.78, 20.54); the pooled NLR was 0.42 (95%CI: 0.19, 0.92); the pooled DOR was 14.48 (95%CI: 2.02, 103.94), and the pooled AUC was 0.87 (95%CI: 0.84, 0.90). For the BISAP, the pooled sensitivity was 0.63 (95%CI: 0.23, 0.90); the pooled specificity was 0.93 (95%CI: 0.89, 0.96); the pooled PLR was 8.97 (95%CI: 3.98, 20.19); the pooled NLR was 0.40 (95%CI: 0.13, 1.19); the pooled DOR was 22.39 (95%CI: 3.64, 137.79), and the pooled AUC was 0.93 (95%CI: 0.91, 0.95). A significant difference was found in the pooled AUC between the Ranson and BISAP (*P* = 0.001) (Table 3, Fig 5, S4 Fig).

### Predictive performance of the Ranson and BISAP for ICU admission

**Fig 6:**
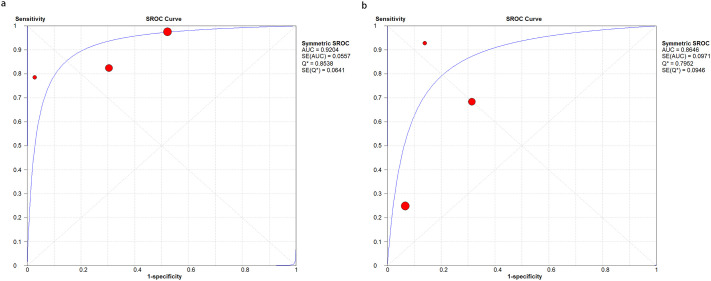
SROC curve of the (a) Ranson and (b) BISAP for predicting ICU admission in AP. SROC, Summary receiver operating characteristic; BISAP, Bedside Index of Severity in Acute Pancreatitis; ICU, intensive care unit; AP, acute pancreatitis.

Three studies [25, 31, 34] were identified for ICU admission. A “shoulder-arm” distribution was demonstrated in the SROC curve for the Ranson, indicating the existence of a threshold effect. The Spearman correlation coefficient for the Ranson was 1.000 (*P* = 0.000), which further confirmed the presence of threshold effects. No “shoulder-arm” distribution and a Spearman correlation coefficient of 0.5 (*P* = 0.667) for the BISAP suggested that no threshold effect existed (Fig 6).

For the Ranson, the pooled sensitivity was 0.86 (95%CI: 0.77, 0.92); the pooled specificity was 0.58 (95%CI: 0.55, 0.61); the pooled PLR was 2.93 (95%CI: 1.43, 6.00); the pooled NLR was 0.23 (95%CI: 0.14, 0.38); the pooled DOR was 26.80 (95%CI: 5.31, 135.23), and the pooled AUC was 0.92 (95%CI: 0.81, 1.00). For the BISAP, the pooled sensitivity was 0.63 (95%CI: 0.52, 0.73); the pooled specificity was 0.84 (95%CI: 0.81, 0.86); the pooled PLR was 3.50 (95%CI: 1.70, 7.24); the pooled NLR was 0.47 (95%CI: 0.21, 1.07); the pooled DOR was 7.68 (95%CI: 2.49, 23.74), and the pooled AUC was 0.86 (95%CI: 0.67, 1.00). No significant difference was found in the pooled AUC between the Ranson and BISAP (*P* = 0.592) (Table 3, Fig 6, S5 Fig).

### Publication bias assessment

Publication bias was evaluated for the outcome morality. The Deeks’ funnel plot asymmetry test showed that there was no publication bias for the Ranson (T = -0.66, *P* = 0.525) (S6 Fig), and for the BISAP, publication bias may also not exist (T = 2.23, *P* = 0.05) (S7 Fig).

## Discussion

This meta-analysis comprehensively assessed and compared the performance of the Ranson and BISAP in predicting the severity and prognosis of AP for the first time. The combined results illustrated that the Ranson had a similar predictive capability to the BISAP; the sensitivity of the Ranson was higher than that of the BISAP, while the BISAP showed higher specificity, PLR and NLR than the Ranson in general.

Chandra et al. [16] conducted a systematic review to estimate the predictive performance of the BISAP for severe AP, and showed that the BISAP had good predictive value under the revised Atlanta Classification (AUC = 0.92, 95%CI: 0.90, 0.95). A previous meta-analysis also measured the predictive ability of the BISAP for the severity of AP, and the BISAP exhibited low sensitivity (0.65, 95%CI: 0.54, 0.74) but high specificity (0.84, 95%CI: 0.70, 0.92) [17]. In another meta-analysis by Gao et al. [18], the BISAP was identified as a reliable in predicting mortality and severity in AP, and in contrast to the Ranson, the BISAP had lower sensitivity and greater specificity. The current meta-analysis specifically investigated and compared the predictive value of the Ranson and BISAP for the severity and prognosis (mortality, organ failure, pancreatic necrosis, and ICU admission) of AP patients, in order to assess the current applicability of the Ranson score in the clinical practice.

As regards prediction of severity and prognosis in AP, the Ranson exhibited a comparable AUC to the BISAP, suggesting that the Ranson and BISAP had an equivalent performance in general. Further, it was found that the Ranson had greater sensitivity but lower specificity than the BISAP. These findings were partially supported by Gao et al. [18] and Papachristou et al. [38], and indicated that the Ranson was an applicable tool for predicting severity and prognosis of patients with AP, with reliable diagnostic accuracy, although its specificity needs to be improved in the future. Mikó et al. [13] also reported similar predictive value of the Ranson and BISAP for mortality and severity of AP. Notably, the Ranson had some important strengths. First, an increase in fluid sequestration within 2 days after admission was evidently associated with persistent organ failure specific to severe AP [39–41], and the factor fluid sequestration is unique to the Ranson score. Second, the Ranson score consists of 11 clinical and laboratory parameters, which allows a more comprehensive consideration of patients, thus increasing the predictive reliability. As for the BISAP, despite 5 parameters required at admission, which simplifies the score assessment, collection of imaging data depends on the physical condition of patients and medical equipment in the hospital. This fact makes it impossible to obtain imaging data from each patient. Third, parameters at 48 hours after admission can reflect dynamic changes in patients’ condition. Evidence has shown that 48-hour variables required by the Ranson enhanced its prognostic accuracy [42, 43]. Based on the above and considering the practicality of the Ranson score in the context of limited resources and time, the Ranson can still be applied in current clinical practice.

The present meta-analysis included 17 studies [21–37] with 5476 AP patients, and evaluated the predictive performance of the Ranson versus BISAP for severity, mortality, organ failure, pancreatic necrosis, and ICU admission in AP, based on the latest Atlanta Classification and Definitions in 2012. Publication bias may not exist, indicating the stability of the results to some extent. According to the findings, clinicians may adopt the Ranson score to predict disease severity and outcomes for patients with AP, and provide personalized counselling and intervention strategies for better management of AP and prognosis improvement of patients. Several limitations should be noted in this study. Firstly, the results showed high heterogeneity. Different etiologies may increase heterogeneity, but due to the mixed etiologies in most patients, subgroup analysis based on etiology could not be achieved. Besides, age may affect heterogeneity, and evidence has shown that the BISAP can effectively predict the severity, pancreatic necrosis, and death of elderly patients with acute pancreatitis, and the Ranson is more effective in assessing the severity of young patients [44], but all the included studies reported mixed ages, which made further subgroup analysis impossible. Secondly, the majority of the included studies were performed in Asia, and the lack of research on other regions may limit the extrapolation of our results. Thirdly, the number of the included studies was small for some outcomes, potentially affecting the stability of the results.

## Conclusion

The Ranson score exhibited a similar predictive performance to the BISAP, and had greater sensitivity but lower specificity than the BISAP, indicating the applicability and accuracy of the Ranson in predicting severity and prognosis of AP patients under limited resources and time. More large-scale studies are warranted to confirm these findings.

## Supporting information

S1 Fig Sensitivity and specificity of the (a) Ranson and (b) BISAP for predicting severity in AP. BISAP, Bedside Index of Severity in Acute Pancreatitis; AP, acute pancreatitis.
(TIF)

S2 Fig Sensitivity and specificity of the (a) Ranson and (b) BISAP for predicting mortality in AP. BISAP, Bedside Index of Severity in Acute Pancreatitis; AP, acute pancreatitis.
(TIF)

S3 Fig Sensitivity and specificity of the (a) Ranson and (b) BISAP for predicting organ failure in AP. BISAP, Bedside Index of Severity in Acute Pancreatitis; AP, acute pancreatitis.
(TIF)

S4 Fig Sensitivity and specificity of the (a) Ranson and (b) BISAP for predicting pancreatic necrosis in AP. BISAP, Bedside Index of Severity in Acute Pancreatitis; AP, acute pancreatitis.
(TIF)

S5 Fig Sensitivity and specificity of the Ranson and BISAP for predicting ICU admission in AP. (a) Sensitivity of the Ranson; (b) Specificity of the Ranson; (c) Sensitivity of the BISAP; (d) Specificity of the BISAP. BISAP, Bedside Index of Severity in Acute Pancreatitis; ICU, intensive care unit; AP, acute pancreatitis.
(TIF)

S6 Fig Publication bias assessment of the Ranson for morality in AP. AP, acute pancreatitis.
(TIF)

S7 Fig Publication bias assessment of the BISAP for morality in AP. AP, acute pancreatitis.
(TIF)

S1 Checklist PRISMA 2020 checklist. (DOCX)

S1 Dataset Minimal data set. (DOCX)
