# Supplementary figures and images for: Predictive value of the Ranson and BISAP scoring systems for the severity and prognosis of acute pancreatitis: A systematic review and meta-analysis
Source: PLoS One. 2024 Apr 30;19(4):e0302046. doi: 10.1371/journal.pone.0302046 (PMC11060534; doi:10.1371/journal.pone.0302046)

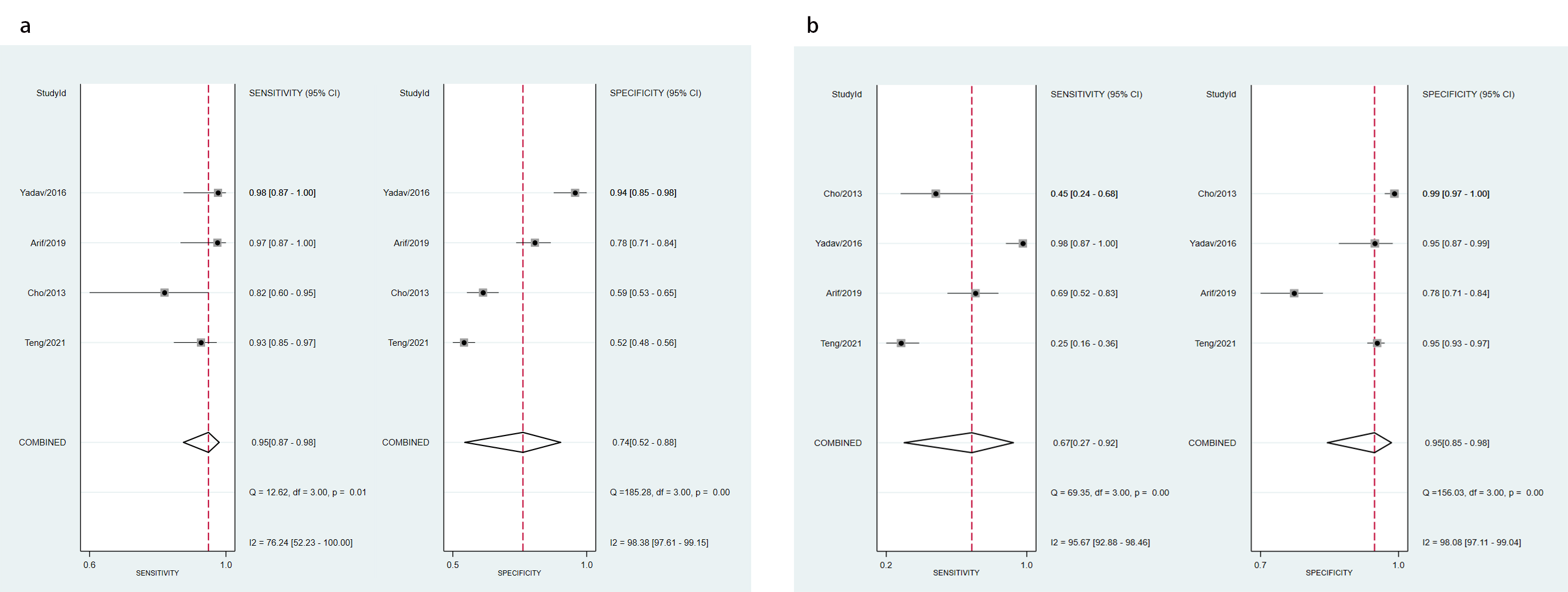

Supplement: S1 Fig — BISAP, Bedside Index of Severity in Acute Pancreatitis; AP, acute pancreatitis. (TIF) [file pone.0302046.s001.tif]

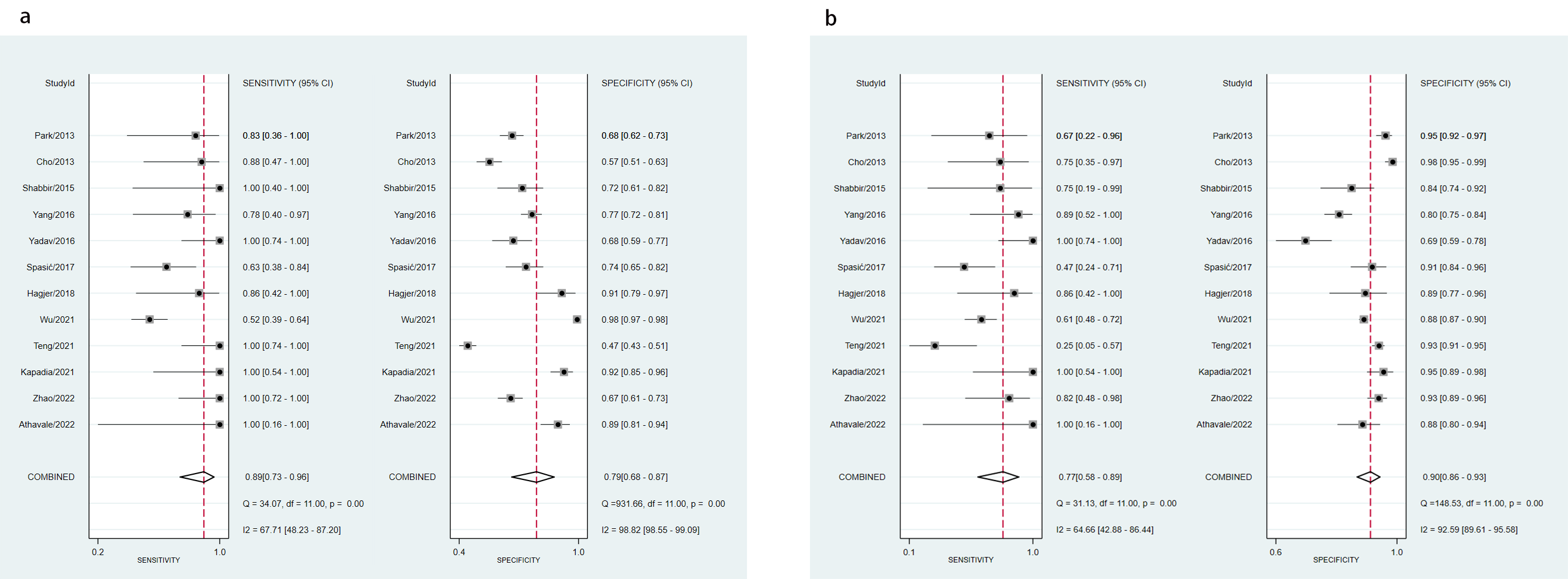

Supplement: S2 Fig — BISAP, Bedside Index of Severity in Acute Pancreatitis; AP, acute pancreatitis. (TIF) [file pone.0302046.s002.tif]

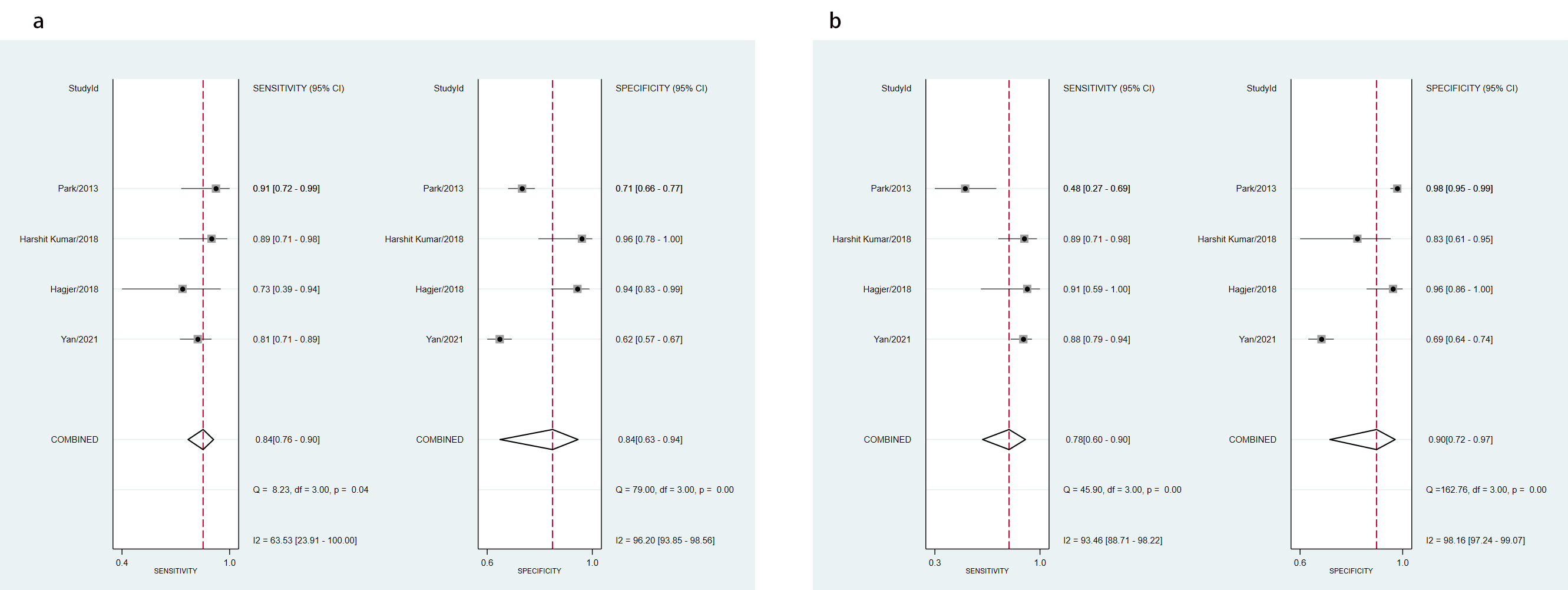

Supplement: S3 Fig — BISAP, Bedside Index of Severity in Acute Pancreatitis; AP, acute pancreatitis. (TIF) [file pone.0302046.s003.tif]

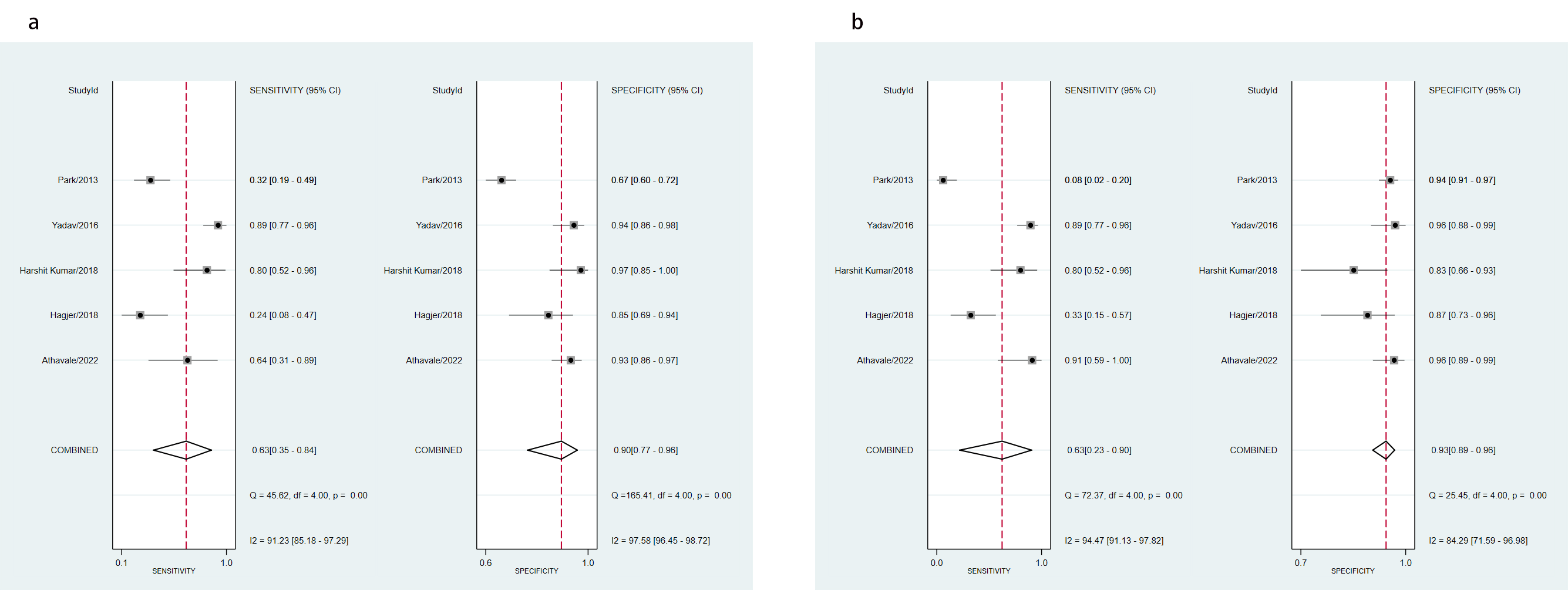

Supplement: S4 Fig — BISAP, Bedside Index of Severity in Acute Pancreatitis; AP, acute pancreatitis. (TIF) [file pone.0302046.s004.tif]

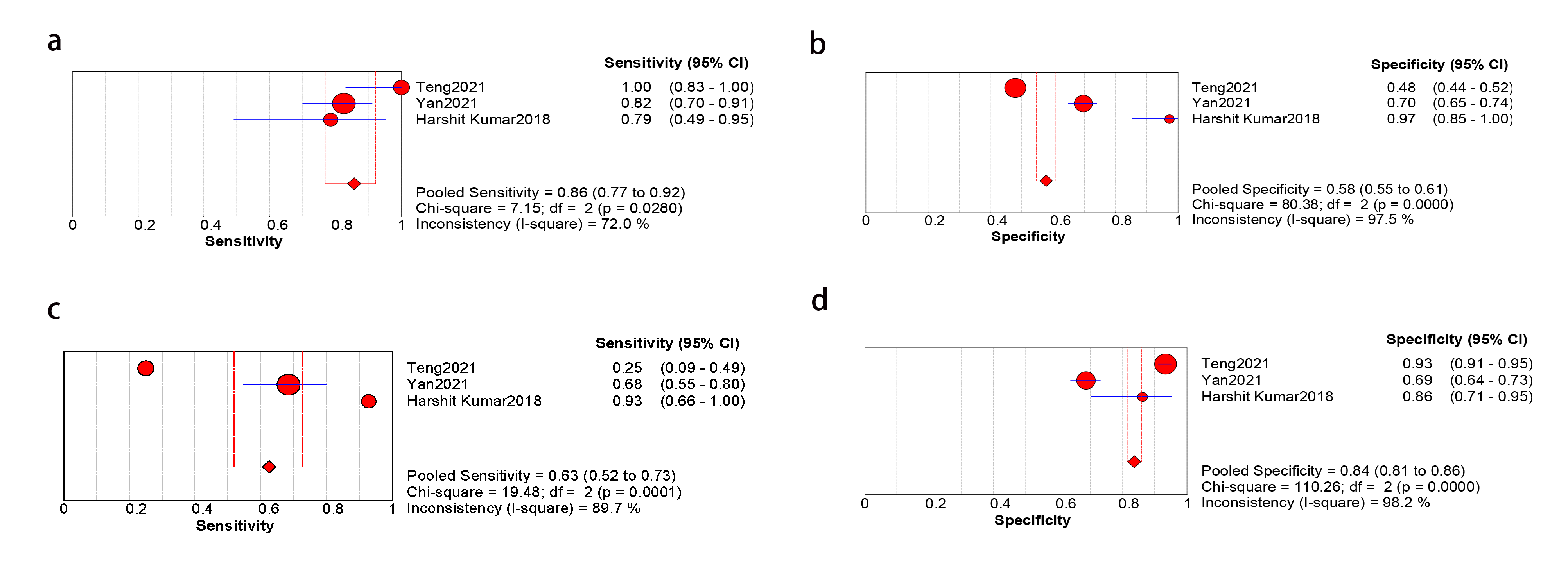

Supplement: S5 Fig — (a) Sensitivity of the Ranson; (b) Specificity of the Ranson; (c) Sensitivity of the BISAP; (d) Specificity of the BISAP. BISAP, Bedside Index of Severity in Acute Pancreatitis; ICU, intensive care unit; AP, acute pancreatitis. (TIF) [file pone.0302046.s005.tif]

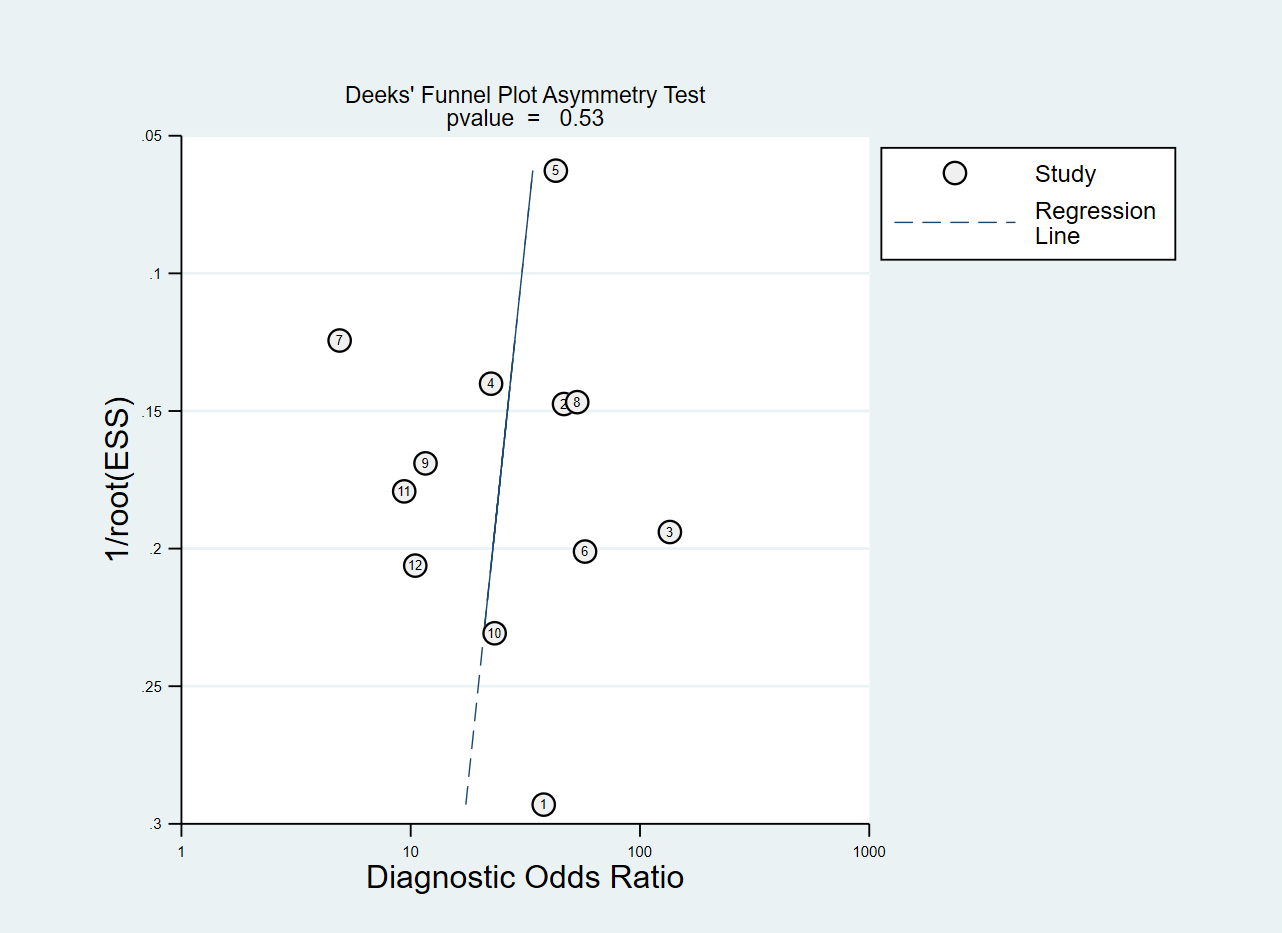

Supplement: S6 Fig — AP, acute pancreatitis. (TIF) [file pone.0302046.s006.tif]

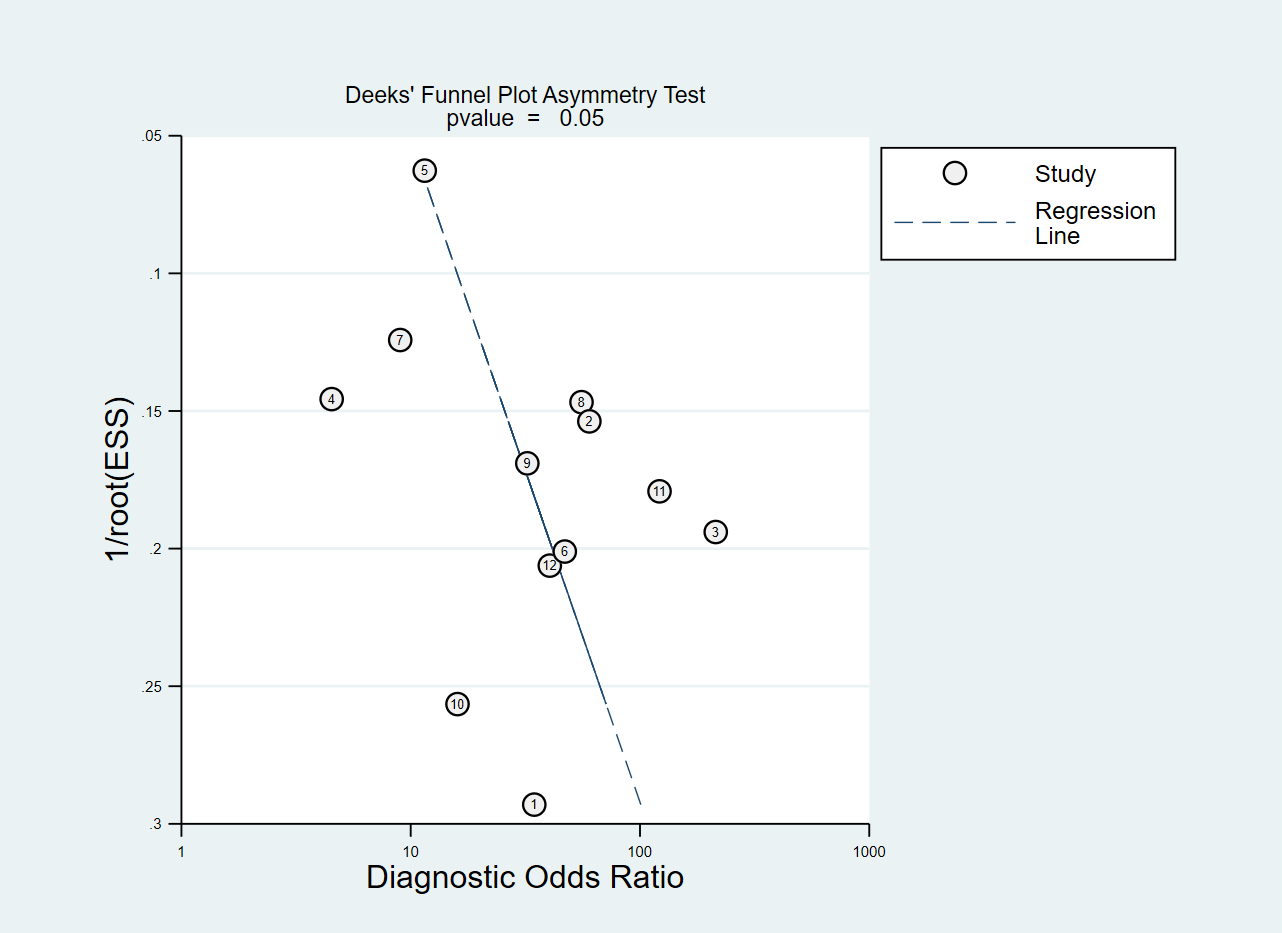

Supplement: S7 Fig — AP, acute pancreatitis. (TIF) [file pone.0302046.s007.tif]
