# Supplementary material for: Predictive value of the Ranson and BISAP scoring systems for the severity and prognosis of acute pancreatitis: A systematic review and meta-analysis
Source: PLoS One. 2024 Apr 30;19(4):e0302046. doi: 10.1371/journal.pone.0302046 (PMC11060534; doi:10.1371/journal.pone.0302046)
Supplement: S1 Dataset — (DOCX) [file pone.0302046.s009.docx]

Minimal Data Set

| Author | Year | Setting | Group | TP | FP | FN | TN |
| --- | --- | --- | --- | --- | --- | --- | --- |
| Severe acute pancreatitis | | | | | | | |
| Teng | 2021 | Singapore 2009-2016 | BISAP≥3 | 20 | 27 | 61 | 545 |
| Teng | 2021 | Singapore 2009-2016 | Ranson≥3 | 75 | 275 | 6 | 297 |
| Arif | 2019 | Pakistan 2015 | BISAP≥3 | 27 | 37 | 12 | 130 |
| Arif | 2019 | Pakistan 2015 | Ranson≥3 | 38 | 36 | 1 | 131 |
| Yadav | 2016 | India 2012-2014 | BISAP≥3 | 41 | 4 | 1 | 73 |
| Yadav | 2016 | India 2012-2014 | Ranson≥3 | 41 | 5 | 1 | 72 |
| Cho | 2013 | Korea 2008-2010 | BISAP≥3 | 10 | 3 | 12 | 274 |
| Cho | 2013 | Korea 2008-2010 | Ranson≥3 | 18 | 113 | 4 | 163 |
| Mortality | | | | | | | |
| Athavale | 2022 | India 2020-2022 | BISAP≥3 | 2 | 12 | 0 | 86 |
| Athavale | 2022 | India 2020-2022 | Ranson≥3 | 2 | 11 | 0 | 87 |
| Zhao | 2022 | China, Shanghai 2018-2020 | BISAP≥3 | 9 | 19 | 2 | 254 |
| Zhao | 2022 | China, Shanghai 2018-2020 | Ranson≥3 | 11 | 90 | 0 | 183 |
| Kapadia | 2021 | Pakistan, Karachi 2017-2018 | BISAP≥3 | 6 | 7 | 0 | 123 |
| Kapadia | 2021 | Pakistan, Karachi 2017-2018 | Ranson≥3 | 6 | 11 | 0 | 119 |
| Teng | 2021 | Singapore 2009-2016 | BISAP≥3 | 3 | 44 | 9 | 597 |
| Teng | 2021 | Singapore 2009-2016 | Ranson≥3 | 12 | 338 | 0 | 303 |
| Wu | 2021 | Nanning 2003-2020 | BISAP≥3 | 40 | 210 | 26 | 1572 |
| Wu | 2021 | Nanning 2003-2020 | Ranson≥3 | 34 | 43 | 32 | 1739 |
| Hagjer | 2018 | India 2015-2016 | BISAP≥3 | 6 | 6 | 1 | 47 |
| Hagjer | 2018 | India 2015-2016 | Ranson≥3 | 6 | 5 | 1 | 48 |
| Spasić | 2017 | Serbia 2011-2014 | BISAP≥3 | 9 | 10 | 10 | 100 |
| Spasić | 2017 | Serbia 2011-2014 | Ranson≥3 | 12 | 28 | 7 | 80 |
| Yadav | 2016 | India 2012-2014 | BISAP≥3 | 12 | 33 | 0 | 74 |
| Yadav | 2016 | India 2012-2014 | Ranson≥3 | 12 | 34 | 0 | 73 |
| Yang | 2016 | Beijing 2007-2015 | BISAP≥3 | 8 | 63 | 1 | 254 |
| Yang | 2016 | Beijing 2007-2015 | Ranson≥3 | 7 | 73 | 2 | 242 |
| Shabbir | 2015 | Pakistan 2010 | BISAP≥3 | 3 | 12 | 1 | 64 |
| Shabbir | 2015 | Pakistan 2010 | Ranson≥3 | 4 | 21 | 0 | 55 |
| Cho | 2013 | Korea 2008-2010 | BISAP≥3 | 6 | 7 | 2 | 284 |
| Cho | 2013 | Korea 2008-2010 | Ranson≥3 | 7 | 124 | 1 | 166 |
| Park | 2013 | Seoul 2007-2010 | BISAP≥3 | 4 | 14 | 2 | 283 |
| Park | 2013 | Seoul 2007-2010 | Ranson≥3 | 5 | 96 | 1 | 201 |
| Organ failure | | | | | | | |
| Yan | 2021 | China 2018-2020 | BISAP≥3 | 75 | 117 | 10 | 263 |
| Yan | 2021 | China 2018-2020 | Ranson≥3 | 69 | 143 | 16 | 237 |
| Hagjer | 2018 | India 2015-2016 | BISAP≥3 | 10 | 2 | 1 | 47 |
| Hagjer | 2018 | India 2015-2016 | Ranson≥3 | 8 | 3 | 3 | 46 |
| Harshit Kumar | 2018 | India 2015-2016 | BISAP≥3 | 24 | 4 | 3 | 19 |
| Harshit Kumar | 2018 | India 2015-2016 | Ranson≥3 | 24 | 1 | 3 | 22 |
| Park | 2013 | Seoul 2007-2010 | BISAP≥3 | 11 | 7 | 12 | 273 |
| Park | 2013 | Seoul 2007-2010 | Ranson≥3 | 21 | 80 | 2 | 200 |
| Pancreatic necrosis | | | | | | | |
| Athavale | 2022 | India 2020-2012 | BISAP≥3 | 10 | 4 | 1 | 85 |
| Athavale | 2022 | India 2020-2012 | Ranson≥3 | 7 | 6 | 4 | 83 |
| Hagjer | 2018 | India 2015-2016 | BISAP≥3 | 7 | 5 | 14 | 34 |
| Hagjer | 2018 | India 2015-2016 | Ranson≥3 | 5 | 6 | 16 | 33 |
| Harshit Kumar | 2018 | India 2015-2016 | BISAP≥3 | 12 | 6 | 3 | 29 |
| Harshit Kumar | 2018 | India 2015-2016 | Ranson≥3 | 12 | 1 | 3 | 34 |
| Yadav | 2016 | India 2012-2014 | BISAP≥3 | 42 | 3 | 5 | 69 |
| Yadav | 2016 | India 2012-2014 | Ranson≥3 | 42 | 4 | 5 | 68 |
| Park | 2013 | Seoul 2007-2010 | BISAP≥3 | 3 | 15 | 37 | 248 |
| Park | 2013 | Seoul 2007-2010 | Ranson≥3 | 13 | 88 | 27 | 175 |
| Intensive care unit admission | | | | | | | |
| Teng | 2021 | Singapore 2009-2016 | Ranson≥3 | 20 | 330 | 0 | 303 |
| Teng | 2021 | Singapore 2009-2016 | BISAP≥3 | 5 | 42 | 15 | 591 |
| Yan | 2021 | China 2018-2020 | BISAP≥3 | 39 | 128 | 18 | 280 |
| Yan | 2021 | China 2018-2020 | Ranson≥3 | 47 | 124 | 10 | 284 |
| Harshit Kumar | 2018 | India 2015-2016 | BISAP≥3 | 13 | 5 | 1 | 31 |
| Harshit Kumar | 2018 | India 2015-2016 | Ranson≥3 | 11 | 1 | 3 | 35 |

TP, true positive; FP, false positive; FN, false negative; TN, true negative.
